# Cumulative Impact of HIV and Multiple Concurrent Human Papillomavirus Infections on the Risk of Cervical Dysplasia

**DOI:** 10.1155/2016/7310894

**Published:** 2016-02-22

**Authors:** David H. Adler, Melissa Wallace, Thola Bennie, Beau Abar, Tracy L. Meiring, Anna-Lise Williamson, Linda-Gail Bekker

**Affiliations:** ^1^Department of Emergency Medicine, University of Rochester, Rochester, NY 14642, USA; ^2^Desmond Tutu HIV Centre, Institute of Infectious Diseases & Molecular Medicine, Faculty of Health Sciences, University of Cape Town, Anzio Road, Observatory, Cape Town, South Africa; ^3^Institute of Infectious Diseases & Molecular Medicine and Division of Medical Virology, Faculty of Health Sciences, University of Cape Town, Anzio Road, Observatory, Cape Town, South Africa; ^4^National Health Laboratory Service, Groote Schuur Hospital, Cape Town, South Africa

## Abstract

Infection with HIV is known to increase the risk of cervical cancer. In addition, evidence suggests that concurrent infection with multiple human papillomavirus (HPV) genotypes increases the risk of cervical dysplasia more than infection with a single HPV genotype. However, the impact of the combination of HIV coinfection and presence of multiple concurrent HPV infections on the risk of cervical dysplasia is uncertain. We compared the results of HPV testing and Pap smears between HIV-infected and HIV-uninfected young women to assess the cumulative impact of these two conditions. We found that both HIV and the presence of multiple concurrent HPV infections are associated with increased risk of associated Pap smear abnormality and that the impact of these two risk factors may be additive.

## 1. Introduction

Coinfection with HIV has a significant impact on the natural history of high-risk human papillomavirus (HR-HPV) infections, the causative agents of cervical cancer. Women with HIV are more likely to be infected with HPV, and more likely to harbor multiple concurrent HPV infections (i.e., simultaneous infection with two or more HPV genotypes) [1]. While many HPV infections are transient, HIV-infected women are more likely to have persistent HPV infections [2, 3] which are associated with a greater incidence and progression of the precancerous lesions caused by HPV [4, 5]. Worsening immunodeficiency correlates with an increased risk of advanced cervical dysplasia [6]. Invasive cervical cancer, an AIDS defining illness, is 2 to 22 times more likely to develop in HIV-infected women [7].

Multiple concurrent HPV infections have been associated with an increased risk of persistent HPV infection [8] and of developing precancerous cervical lesions [9, 10] compared to infection with a single HPV genotype. Simultaneous infection with HIV and multiple concurrent HPVs may increase the risk of developing precancerous lesions more than either condition on its own.

Cervical cancer is the number one cancer cause of years of life lost in the developing world [11] where over eighty-five percent of global cases and deaths from cervical cancer occur, with the very highest rates in Sub-Saharan Africa [12, 13]. We compared the prevalence of multiple concurrent high-risk HPV infections between HIV-infected and HIV-uninfected South African young women and the association of these infections with cervical cytological abnormalities.

## 2. Materials and Methods

Between October 2013 and March 2015, we conducted a longitudinal study of 50 HIV-infected and 50 HIV-uninfected young, sexually active, South African women. Cohort enrollment occurred sequentially until the goal of 50 participants in each group was met. Study participants were recruited through the Youth Centre in Masiphumelele and the Hannan Crusaid Clinic in Gugulethu, townships in the Cape Town area of South Africa. The Youth Centre in Masiphumelele serves the general adolescent population of the township, while the Hannan Crusaid Clinic specifically serves the HIV-infected population of Gugulethu. Demographic and behavioral variables of our cohort are presented in Table 1. Informed consent (age 18 years or older) or parental consent along with signed adolescent assent (age 16-17 years) was obtained from all participants. The Institutional Review Boards of the University of Rochester and the University of Cape Town approved this study.

Serial HPV DNA testing, utilizing self-collected specimens, was conducted approximately every six months for all participants. In addition, all study participants underwent baseline Pap smear testing. Most study participants underwent additional Pap testing during the follow-up period. Subsequent Pap testing (not included in this analysis) was conducted at varying intervals depending on HIV status and previous Pap result as per the guidelines of the Western Cape Province. Only the enrollment HPV and Pap test results are used in this analysis. HIV status was confirmed upon enrollment and HIV-uninfected participants underwent HIV testing at every study visit. Women with a history of HPV vaccination or cervical surgery were excluded.

Roche Diagnostics Linear Array HPV Test was used for all HPV genotyping. This kit identifies 37 HPV genotypes including all oncogenic “high-risk” HPV genotypes (HR-HPV) as designated by the International Agency for Research on Cancer [14]. These 13 HR-HPV genotypes include types 16, 18, 31, 33, 35, 39, 45, 51, 52, 56, 58, 59, and 68. Specimens for HPV testing were obtained via self-sampling, performed in private, in which study participants were instructed to twirl a Dacron swab high in the vagina for 10 seconds. Specimens were then transported to the laboratory in Digene transport medium. The MagNA Pure Compact Nucleic Acid Isolation Kit (Roche) was used to extract DNA. All Pap smears were reported per the Bethesda system. Per laboratory protocol a pathologist reviewed all positive Pap smears. We defined any Pap not categorized as “negative,” including atypical squamous cell of undetermined significance (ASCUS) to be abnormal.

Pearson *χ* ^2^ tests for independence were used to compare groups, and Pearson and Spearman correlations were used to examine bivariate associations. All analyses were performed using IBM SPSS 22.0.

## 3. Results

Preliminary descriptive cross-sectional data from the initial 85 participants in this cohort have been previously published [15]. Among our final and complete cohort, we found an overall HPV prevalence rate of 64% and HR-HPV prevalence rate of 38%. Multiple concurrent HPV infections were found among 41% of participants and multiple concurrent HR-HPV infections were found among 24%. Since a wealth of research has shown cytological abnormalities to be driven by the presence of an HR-HPV infection, we examined Pap test results only among women with at least one such infection. The results indicated that having multiple concurrent HPV infections was associated with twice the likelihood of an associated Pap smear abnormality compared to having a single HPV infection (34% versus 17%), although this difference did not reach statistical significance (*p* = 0.15).

Overall, HIV-infected women had a much greater burden of HPV infection. HIV-infected participants had greater proportions of multiple concurrent HPV infections (60% versus 22%, *p* < 0.001) and multiple concurrent HR-HPV infections (36% versus 12%, *p* = 0.004). The average number of HPV infections was 2.92 for the HIV-infected group compared to 0.90 for the HIV-uninfected group (*p* < 0.001). Likewise, the average number of HR-HPV infections was greater among those with HIV (1.16 versus 0.36, *p* = 0.001). HIV-infected participants had a greater proportion of abnormal Pap smears at baseline (24% versus 12%), although this difference was not statistically significant (*p* = 0.12).

Among HIV-infected women only, those with two or more HPV infections were more likely to have an associated Pap abnormality compared to those with zero or one HPV infection (33% versus 10%, *p* = 0.058). When eliminating those women without an HPV infection and comparing HIV-infected women with multiple infections to those with a single HPV infection, the difference is still substantial (33% versus 20%) although not statistically significant (*p* = 0.43). Thus, there may be an additive effect in which the combination of HIV infection and the presence of multiple concurrent HPV infections increases the association with cervical dysplasia greater than either risk factor alone.

Table 2 compares and summarizes the cross-sectional epidemiological differences in HPV and cervical dysplasia between HIV-infected and HIV-uninfected study participants. When focusing on HR-HPV infections, we found that, among HIV-infected women, there was a similar proportion of abnormal Pap tests results for those with one HR-HPV infection (38%) and those with more than one (39%) (*p* = 0.94). Among HIV-uninfected women, the difference is greater (1 HR-HPV infection = 0%, 2 or more HR-HPV infections = 50%) though not statistically significant due to the small size of this subset (*p* = 0.18).

Overall, our HIV-infected participants were not severely immune-compromised. Their average CD4 count was 518/mm^3^ (IQR = 366–598; CD4 counts were not available for 10 participants). Forty-four percent were receiving antiretroviral (ARV) medications at the time of testing. The average CD4 count for those on ARV medications was 482 and for those not on ARV medications was 547 (*p* = 0.37). CD4 count was not found to be significantly associated with abnormal Pap test (*p* = 0.21) or total HPV infections (*p* = 0.07), although higher CD4 counts were associated with fewer HR-HPV infections (*r* = −0.36, *p* = 0.02). No significant relationship between ARV medications and the total number of HPV infections (*p* = 0.54) or the number of high-risk HPV infections (*p* = 0.77) was identified.

Our bivariate analyses identified positive associations between age and number of HPV infections (Pearson *r* = 0.23, *p* = 0.019), number of HR-HPV infections (Pearson *r* = 0.20, *p* = 0.048), and baseline Pap test abnormality (Spearman *r* = 0.20, *p* = 0.050). There were no relationships observed between HPV infections or Pap abnormality and lifetime smoking, lifetime number of sexual partners, number of sexual partners in the last 6 months, and current use of condoms, birth control pills, or birth control injections (all *p* values > 0.10).

## 4. Discussion

Our results demonstrate a large burden of HPV among our study population with a notably high prevalence of multiple concurrent infections. This is consistent with previous data from South Africa [16]. We confirmed previous work that identified a strong positive association between HIV and both HPV infection and HPV-related cervical dysplasia. Moreover, we found that the increased risks for cervical dysplasia conferred by HIV infection and multiple concurrent HPV infection may be additive.

It is well established that HPV causes cervical cancer [17]. It is less clear, however, whether multiple concurrent HPV infections increase this risk. Some research has demonstrated an increased risk of carcinogenesis due to multiple concurrent infections compared to infection with a single HPV genotype [10, 18]. One study identified a linear association between the number of HR-HPV infections and the severity of associated cervical dysplasia [9]. In a study of HIV-infected women, however, multiple concurrent infections were not found to have an increased association with cervical dysplasia [19]. We found a significantly positive association (twice the probability) between multiple concurrent infections and the risk of associated Pap smear abnormality.

Likewise, HIV infection is known to increase the risk of cervical dysplasia [2]. Data from the present study demonstrate HIV-infected participants to have a twofold greater proportion of Pap abnormalities compared to their HIV-uninfected counterparts. Furthermore, when looking only at HIV-infected participants, a large but not statistically significant difference in association with Pap abnormality was found between those with a single HPV infection and those with multiple concurrent HPV infections, indicating a possible additive effect of HIV infections and infection with multiple concurrent HPVs in increasing the risk of cervical dysplasia.

The impact of multiple concurrent HPV infections on the duration of infection is uncertain. Some prior research has found no impact of multiple concurrent infections on HPV persistence [20, 21]. In contrast, in a study of genotype specific duration of HPV infection, investigators found that coinfection with multiple HPV types increased the duration of infection for all HR-HPVs as a group as well as for HPV16 specifically [8]. The persistence of HPV infection is of critical importance as it has been demonstrated to increase the risk for incident precancerous lesions of the cervix [4, 22]. Due to sample size limitations, this study did not assess the impact of multiple concurrent HPV infections on HPV persistence.

This study has several limitations. The generalizability of the findings presented may be limited due to the specific geographical area from which the study cohort was drawn. Despite the numerous statistically significant findings, the relatively small sample size may have restricted the identification of important differences between groups and subgroups. Likewise, multivariate analyses examining differences between HIV-infected and HIV-uninfected participants while controlling for behavioral and demographic variables were not feasible due to the sample size. Lastly, because of the relative immune competency of our HIV-infected group and sample size limitations, study findings cannot be correlated with the extent of immunosuppression among HIV-infected participants.

## 5. Conclusion

In our cohort, Pap smear abnormalities were identified twice as frequently among HIV-infected women as among their HIV-uninfected counterparts. In addition, among all women in the cohort, having multiple concurrent HPV infections was associated with twice the likelihood of an associated Pap smear abnormality compared to having a single HPV infection. These risk factors may be additive given our finding that HIV-infected women with multiple concurrent HPV infections were more likely to have associated Pap smear abnormalities than HIV-infected women with a single HPV infection.

## Figures and Tables

**Table 1:** Participant demographics and behavioral variables.

	HIV-infected				HIV-uninfected				*p*
	M (SD)	IQR	*f*	%	M (SD)	IQR	*f*	%	
Age at first visit	19.6 (1.4)	19.0–21.0			18.4 (1.4)	17.0–19.3			<0.001

Smoker at any time									0.28
No			43	88%			47	94%	
Yes			6	12%			3	6%	

Lifetime sexual partners									0.05
1			11	22%			4	8%	
2–5			34	68%			44	88%	
>5			5	10%			2	4%	

Sexual partners in the last 6 months									0.65
1			48	96%			47	94%	
2–5			2	4%			3	6%	

Number of past pregnancies									0.84
0			33	66%			34	68%	
1			15	30%			15	30%	
2			2	4%			1	2%	

**Table 2:** HPV infections, Pap test results, and HIV status.

	HIV-uninfected		HIV-infected	
	*n* (%)		*n* (%)	
	Normal Pap	Abnormal Pap	Normal Pap	Abnormal Pap
Number of HPV infections				
0	26 (59%)	0 (0%)	10 (26%)	0 (0%)
1	11 (25%)	2 (33%)	8 (21%)	2 (17%)
2	3 (7%)	1 (17%)	4 (11%)	2 (17%)
3	3 (7%)	2 (33%)	6 (16%)	1 (8%)
4	0 (0%)	1 (17%)	2 (5%)	2 (17%)
5	1 (2%)	0 (0%)	2 (5%)	1 (8%)
>5	0 (0%)	0 (0%)	6 (16%)	4 (34%)
Number HR-HPV infections				
0	35 (80%)	3 (50%)	22 (58%)	2 (17%)
1	6 (14%)	0 (0%)	5 (13%)	3 (25%)
2	3 (7%)	3 (50%)	6 (16%)	2 (17%)
3	0 (0%)	0 (0%)	3 (8%)	5 (42%)
4	0 (0%)	0 (0%)	1 (3%)	0 (0%)
5	0 (0%)	0 (0%)	0 (0%)	0 (0%)
>5	0 (0%)	0 (0%)	1 (3%)	0 (0%)

Note: number of HPV infections and number of HR-HPV infections were associated with age (Pearson *r* = 0.23, *p* = 0.019; *r* = 0.20, *p* = 0.048, resp.).
